# Oral Uptake of *Chlamydia psittaci* by Ducklings Results in Systemic Dissemination

**DOI:** 10.1371/journal.pone.0154860

**Published:** 2016-05-11

**Authors:** Simon Thierry, Fabien Vorimore, Christelle Rossignol, Sabine Scharf, Konrad Sachse, Patricia Berthon, Benoit Durand, Isabelle Virlogeux-Payant, Nicole Borel, Karine Laroucau

**Affiliations:** 1 ANSES, Animal Health Laboratory, Bacterial Zoonoses Unit, Maisons-Alfort, France; 2 INRA, UMR1282 Infectiology and Public Health, Nouzilly, France; 3 UMR1282 Infectiology and Public Health, François Rabelais University, Tours, France; 4 Friedrich-Loeffler-Institut (Federal Research Institute for Animal Health), OIE Reference Laboratory for Chlamydiosis, Jena, Germany; 5 ANSES, Animal Health Laboratory, Epidemiology Unit, Maisons-Alfort, France; 6 Institute of Veterinary Pathology, University of Zurich-Vetsuisse, Zurich, Switzerland; Univ. of Texas Health Science Center at San Antonio, UNITED STATES

## Abstract

Enteric infections caused by *Chlamydia (C*.*) psittaci* are frequent in ducks, but mostly remain subclinical under field conditions. To emulate natural infection, we investigated the pathogenic potential of a *C*. *psittaci* field strain in orally inoculated 4-day-old ducklings. Three different challenge doses were tested and seven contact animals were also mock-inoculated with buffer in each group. Over the course of ten days, the birds were monitored for clinical symptoms and chlamydial dissemination before final examination of tissues using histopathology and immunohistochemistry. While the challenge strain disseminated systemically to all internal organs, mild signs of diarrhea were confined to ducklings inoculated with the highest dose (4.3 x 10^8^ IFU/mL, Group 1). No other clinical symptoms or histopathological lesions were seen. The chlamydial load in internal organs as measured by PCR depended on the challenge dose and was unevenly distributed, i.e. high loads in spleen, liver, and distal small and large intestinal tract (ileum, cecum and rectum) vs. ten times lower values in lungs and proximal small intestinal tract (duodenum and jejunum). Notably, the *C*. *psittaci* infection of contact birds became evident on day 10 post-infection, with bacterial loads comparable to those of experimentally-infected animals, thus suggesting rapid bird-to-bird transmission of the challenge strain.

## Introduction

The family *Chlamydiaceae* comprises a group of obligate intracellular Gram-negative bacteria that are widely distributed throughout the world, causing a wide range of diseases in humans and animals [1]. It is composed of a single genus, *Chlamydia*, known to contain 11 species: *C*. *abortus*, *C*. *avium*, *C*. *caviae*, *C*. *felis*, *C*. *gallinacea*, *C*. *muridarum*, *C*. *pecorum*, *C*. *pneumoniae*, *C*. *psittaci*, *C*. *suis*, and *C*. *trachomatis* [2,3].

Avian chlamydiosis due to *C*. *psittaci* is a major factor of economic loss within the poultry industry, and a permanent risk for zoonotic transmission to humans [4,5]. Infection was observed in more than 467 species of birds belonging to 30 different orders [6], including poultry. In birds, symptoms depend on the virulence of the chlamydial strain and sensitivity of the host [7]. In general, transmission occurs through inhalation of aerosolized respiratory secretions or ingestion of contaminated dust.

In turkeys and sometimes in chickens, *C*. *psittaci* infections can lead to respiratory symptoms, a drop in daily weight gains and death, all of which impair the farm’s economic performance [7,8]. To investigate the underlying processes, various experimental models of respiratory infections in turkeys or chickens have been developed [9–11]. In contrast, enteric chlamydial infections due to *C*. *psittaci* occur frequently in ducks, but mostly remain subclinical [12–15], making this infection primarily a public health issue. The initial factors and clinical course of duck infections under field conditions are poorly characterized. The interaction of horizontal and vertical transmission pathways in the infectious process is unknown, but the massive *C*. *psittaci* fecal shedding detected in adult animals on grassland [14,15] strongly suggests indirect transmission through environmental contamination, mainly by the fecal-oral route.

To assess the virulence of *C*. *psittaci* in ducks in light of the known zoonotic potential for humans, infection models emulating natural transmission pathways can be very helpful. For this purpose, an experimental study on ducklings using the oral inoculation route was conducted in order to mimic field conditions and, in particular, investigate asymptomatic carriage and possible inter-animal transmission of *C*. *psittaci*.

## Materials and Methods

### Inoculum

*C*. *psittaci* strain 06–859 (genotype E/B), isolated from a cloacal swab of an asymptomatic mule duck [16], was used as an inoculum. The strain was first passaged four times by injection into the yolk sac of 7-day-old embryonated specific-pathogen-free (SPF) chicken embryos. Once the embryos had died, the vitelline membranes from ten eggs were harvested, ground with sterile glass beads in 2SP medium (sucrose phosphate 2X with 0.1 mg/mL of vancomycin, 0.01 mg/mL of gentamycin, 0.025 mg/mL of amphotericin B and 10% fetal bovine serum) and then centrifuged at 3000 rpm for 10 min. The supernatant was homogenized and stored at -80°C until inoculation. The titer of the inoculum was determined in Buffalo Green Monkey (BGM) cell culture as follows: BGM cells were seeded on round coverslips and cultured in growth medium consisting of minimal essential medium (Sigma-Aldrich, France) supplemented with 5% fetal calf serum (FCS). Tenfold dilutions of the inoculum were centrifuged at 3400 *g* for 1 h at 37°C and incubated for 2 h at 37°C. The growth medium was subsequently replaced by Ultra-MDCK serum-free medium (Ozyme, France). After 48 h of incubation, the cells were fixed in absolute methanol for 10 min. Chlamydial inclusions were detected by direct immunofluorescence using a monoclonal antibody conjugated to fluorescein isothiocyanate (FITC), diluted 1:5 in phosphate buffered saline (PBS), pH 7.4 (Imagen Chlamydia, Oxoid, France). The number of inclusion-forming units (IFU) per mL was assessed by counting the total number of inclusions on the whole coverslip of a countable inoculum dilution. A final titer of 4.3 x 10^8^ IFU/mL was determined for the inoculum.

### Animals

**Fig 1:**
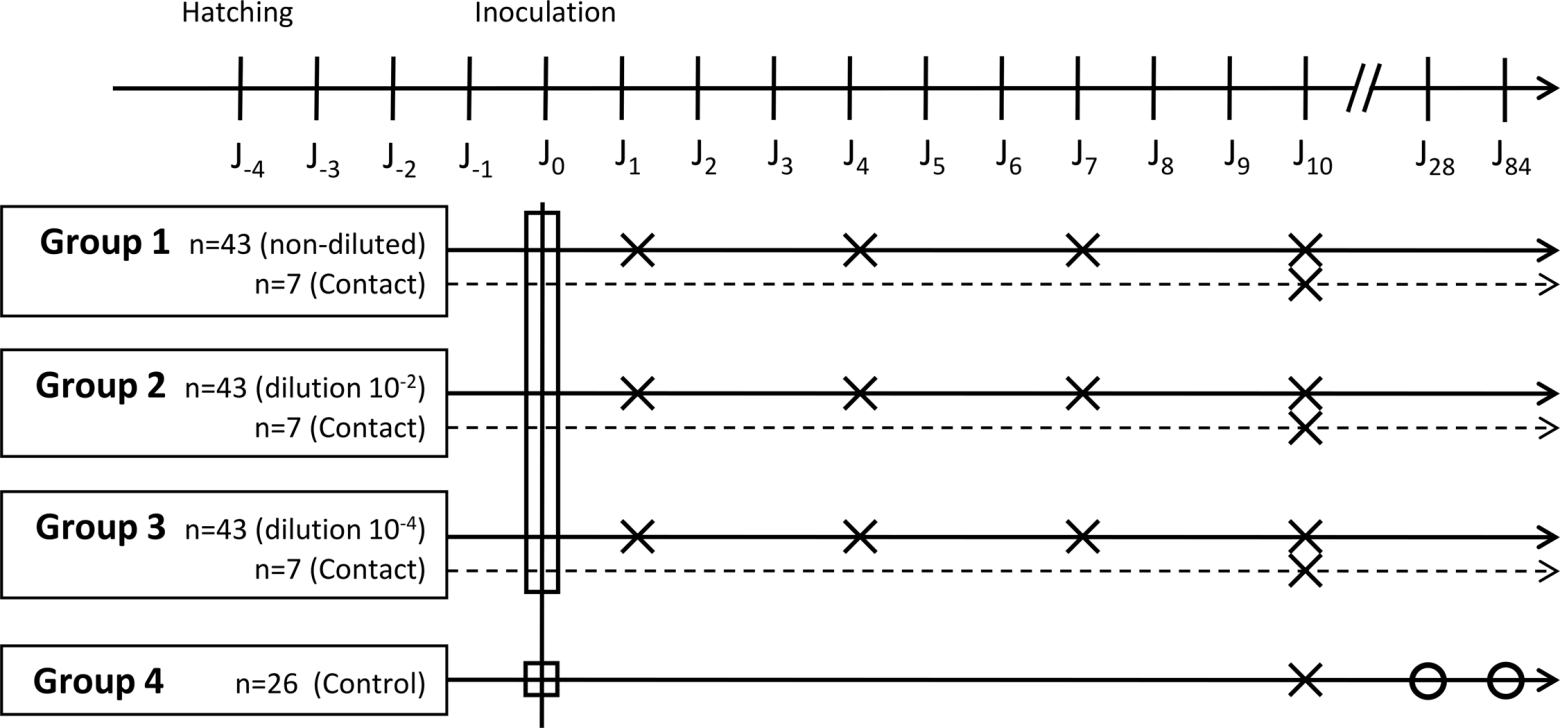
Study design. Circles represent the time points at which five animals were swabbed. Squares and crosses represent the time points at which 14 and seven animals were sacrificed, respectively.

Four-day-old naturally raised (non-SPF) mule ducklings from a single flock were used in this study (n = 176), since SPF mule ducks are unavailable. The parental flock was tested by PCR [17] just after laying the eggs from which ducklings included in this study were hatched. Cloacal swabs from 30 randomly selected females in this flock tested negative for *Chlamydiaceae*. Upon arrival at the Experimental Infectious Disease Platform (PFIE) of the French Institute for Agricultural and Food Research (INRA, Tours, France), ducklings were randomly divided into four groups: three challenge groups (n = 50 per group) with different inoculum doses (Group 1 = non-diluted *C*. *psittaci* inoculum; Group 2 = 10^−2^ dilution; Group 3 = 10^−4^ dilution) and a control group (n = 26; Group 4) (**Fig 1**).

Groups 1 to 3 were housed in three separate 5 m^2^ units under negative pressure with inlet and exhaust HEPA-filtered air, and maintained on a natural light–dark cycle within the biosafety level 3 (BSL 3) animal facility of the PFIE. Group 4 was housed in a 5 m^2^ unit in a BSL 1 animal facility. Antibiotic-free feed and water were provided *ad libitum*.

### Experimental design

The study was approved by the local animal experimentation ethics committee (Comité éthique en expérimentation animale du Val de Loire, n°2010/2). An overview of the experimental design is given in **Fig 1**.

Before inoculation, 14 ducklings (two from each inoculated group and eight from the control group) were sacrificed by cervical dislocation, swabbed and necropsied in order to check by PCR the eventual dissemination of *C*.*psittaci* in these birds coming from a non-SPF flock.

In Groups 1 to 3, 41 birds per group were orally inoculated either with the non-diluted inoculum, or 10^−2^ or 10^−4^ dilutions of the inoculum extemporaneously prepared in PBS, respectively. Doses from previous experimental infections [18,19] served as the basis for our experimental design. The inoculation was performed by orally injecting 200 μL of inoculum into the esophagus using a syringe with a flexible tube. Seven contact birds in each inoculated group were ring-identified and orally inoculated with 200 μL of PBS. These birds were integrated and kept in contact with *C*. *psittaci*-inoculated birds within each group.

Control group birds (Group 4, n = 18) were orally mock-inoculated with 200 μL of PBS.

Seven birds from each infected group (Groups 1 to 3) were sequentially sacrificed by cervical dislocation on 1, 4, 7 or 10 days post-inoculation (dpi). The contact birds from each group were sacrificed on 10 dpi, as were seven birds from the control group. The remaining control group birds were subjected to cloacal swabs on 28 and 84 dpi. Throughout the study period, clinical signs were recorded daily.

### Sample collection

Prior to necropsy, a cloacal swab was taken from each sacrificed animal. Tissue samples from the spleen, liver, lungs and different parts of the intestine (duodenum, jejunum, ileum, ceca and rectum) were collected for PCR and/or culture analysis and weighed before storage at—20°C.

Pieces of spleen, lung and cecum collected on 10 dpi from either birds in the control group or from Group 1 (infected and contact birds) were fixed in formalin (4% buffered neutral formaldehyde), processed, embedded in paraffin, sectioned at 2.5 μm, and stained with hematoxylin and eosin (HE) for histopathological examination.

### Real-time PCR for *Chlamydiaceae*

DNA from the organ samples (spleen, liver, lung, and intestine) was extracted using the QIAmpDNA Mini Kit according to the tissue protocol (Qiagen, Courtaboeuf, France) and eluted in 200 μL of elution buffer. Cloacal swabs were extracted according to the swab protocol and DNA was eluted in 150 μL of elution buffer. DNA extracts were analyzed using the real-time PCR method of Ehricht and colleagues [17]. Each PCR run included serial dilutions of DNA with known genome copies of *C*. *psittaci*, allowing quantification of the template DNA and conversion into the number of *C*. *psittaci* genome copies. The latter was used to calculate *C*. *psittaci* genome copy numbers per gram of organ (Nb of copies/g) according to the weight of each organ sample. For the swab samples, the final results were displayed as the number of *C*. *psittaci* genome copies per swab (Nb of copies/sample). All Cq values above 40 were considered negative.

### Chlamydial replication by re-isolation and genotyping

To assess the viability of the bacteria detected in tissue samples by PCR, the ceca of all animals from Group 1 sacrificed on 10 dpi were cultured in the yolk sacs of embryonated SPF chicken eggs. Samples were ground using sterile glass beads in 2SP medium and then centrifuged at 3000 rpm for 10 min. Supernatants were collected and supplemented with 1 mg/mL of vancomycin, 1 mg/mL of streptomycin, 1 mg/mL of kanamycin and 1000 unit/mL of nystatin then incubated at + 4°C for 20 min. Suspensions were then inoculated into the yolk sacs of 5 embryonated SPF chicken eggs. The eggs were monitored daily and the vitelline membranes of dead embryos were analyzed by PCR as described above.

The *ompA* gene of each re-isolate was partially sequenced using primer pair 5GPF and 3GPB, defining a 1,317 bp segment [20].

### Immunohistochemistry (IHC) for chlamydial antigen detection in tissues

The presence of chlamydial antigen in paraffin sections was examined using a *Chlamydiaceae*-family-specific mouse monoclonal antibody targeting the chlamydial lipopolysaccharide (LPS, Clone ACI-P, Progen, Heidelberg, Germany). After deparaffinization in xylene and rehydration through graded ethanol to water, the antigen was retrieved by heat-induced epitope treatment (citrate buffer, pH 6.0, 98°C for 20 min). After the primary antibody (dilution 1:200) was incubated for 1 h, the endogenous peroxidase activity was inhibited with peroxidase-blocking solution at room temperature (RT) for 5 min. The EnVision Mouse kit (Dako, Glostrup, Denmark) was used as a secondary antibody for 30 min, followed by the 3-amino, 9-ethyl-carbazole (AEC) substrate solution for 10 min. Sections were then counterstained with hematoxylin. A negative control for each section was included using only the antibody diluent instead of the primary antibody.

### Statistical analysis

In inoculated animals, the association between genome copy number, sample type (organ or swab) and inoculation dose was separately analyzed in animals euthanized on 1, 4, 7 or 10 dpi using four linear mixed models. The dependent variable was the genome copy number (on a log10 scale). Constant parameters consisted of the sample type (swab or one of the eight studied organs) and the inoculation dose (qualitative variable: non-diluted, 10^−2^ dilution and 10^−4^ dilution).

In each group, the genome copy number obtained on 10 dpi in inoculated animals was compared to that of contact animals using a mixed linear model. The dependent variable was the genome copy number (on a log10 scale). Constant parameters were the sample type (swab or one of the eight studied organs), whether the animal had been inoculated or not (binary variable), and the inoculation dose (for inoculated animals: the inoculation dose, for contact animals: the dose inoculated to the ducklings with which they were in contact, using the same 3-class coding as above).

## Results

### Clinical signs and macroscopic lesions

Mild diarrhea noted by visual observation of the floor was observed in Group 1 (highest challenge dose), whereas no clinical signs were recorded in the inoculated birds and their corresponding contact birds from Groups 2 and 3. Non-infected birds from Group 4 (control group) remained symptom-free throughout the whole study period. There was no difference in behavior and food intake between the *C*. *psittaci*-inoculated birds and the controls.

No macroscopic lesions were observed during necropsy of birds from any of the groups or at any of the study time points (1, 4, 7 and 10 dpi).

### Chlamydia dissemination in host tissue

**Fig 2:**
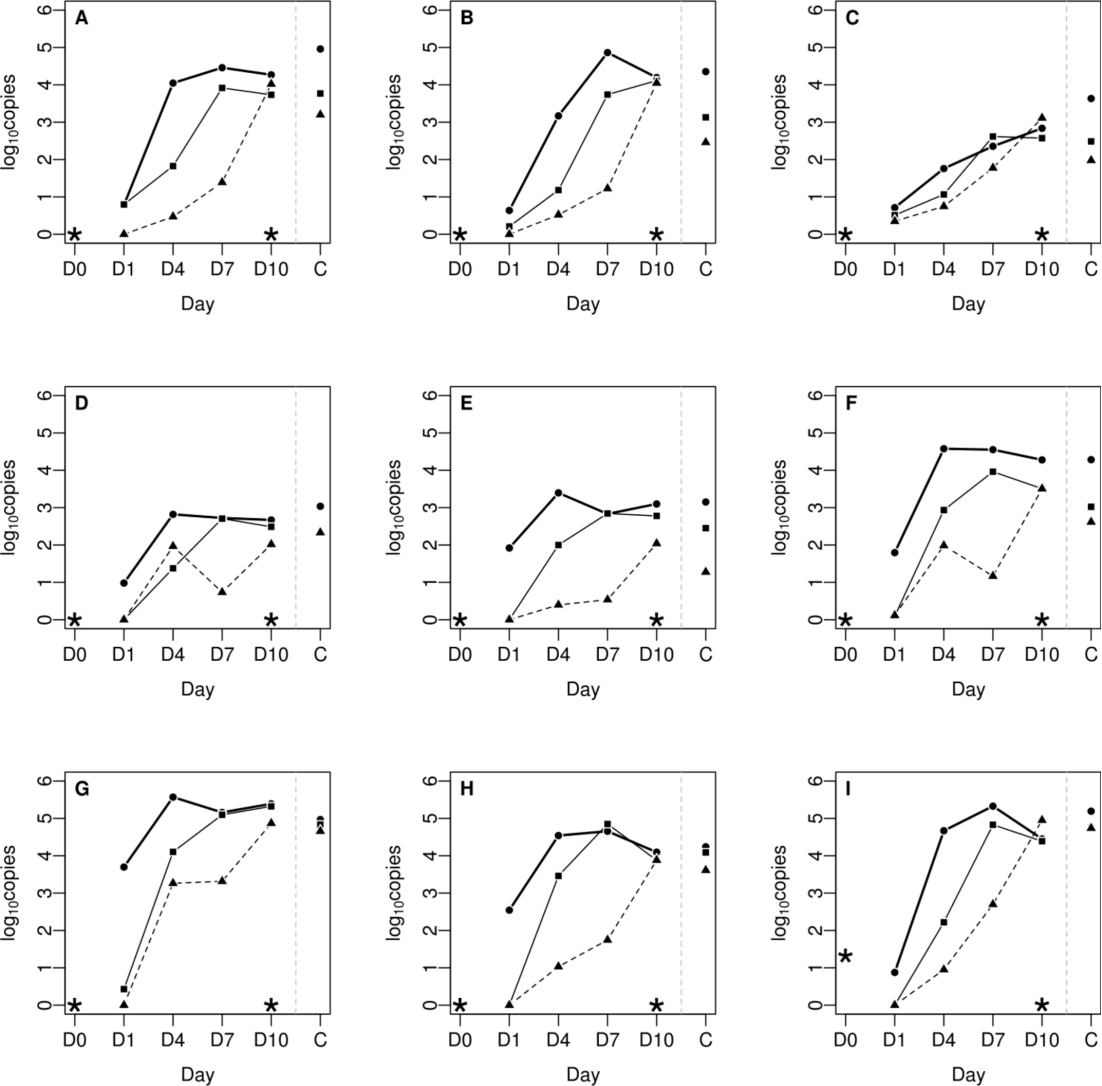
Chlamydial load in 1 g of tissue from individual organs during the course of the 10-day infection experiment (filled circles: non-diluted inoculum; squares: 10^−2^ dilution; triangles: 10^−4^ dilution). A: spleen; B: liver; C: lung; D: duodenum; E: jejunum; F: ileum; G: cecum; H: rectum; I: cloacal swab. (C) Contact birds on 10 dpi. (*) Control group.

In the course of the experimental infection, up until 10 dpi, an increase in the number of *C*. *psittaci* genome copies was observed in all the internal organs of the three challenged groups, suggesting chlamydial replication in the host after oral application (**Fig 2**). A dose effect was observed as birds inoculated with the highest concentration (4.3 x 10^8^ IFU/mL, Group 1) reached the maximum number of genome copies up to 4 dpi, whereas birds inoculated with 4.3 x 10^6^ IFU/mL (Group 2) and 4.3 x 10^4^ IFU/mL (Group 3) peaked at 7 or 10 dpi respectively. In each group, the analysis of the organs from contact birds sacrificed on 10 dpi showed a similar chlamydial load as that observed in challenged birds (P = 0.09).

Comparison between individual organs (spleen, liver, lung, duodenum, jejunum, ileum, cecum, and rectum) and within groups at different time points (1, 4, 7, and 10 dpi) revealed an uneven distribution of chlamydial load (**Fig 2**). High loads were detected in the spleen, liver and the distal small (ileum) and large intestinal tract (cecum and rectum), whereas values were ten times lower in the lungs and proximal small intestinal tract (duodenum and jejunum). The cecum was the organ with the highest chlamydial load (P < 0.001) in all infected groups, including contact birds. The chlamydial dissemination in organs was dose dependent, except for the lungs. A plateau was reached between 4 and 7 dpi, and the level of colonization started to decrease slightly on 10 dpi for the highest doses. The time course for challenge strain dissemination was similar in both inoculated and contact birds, and trends in organ tissue were also comparable.

No *C*. *psittaci* was detected in the organs of control animals sacrificed on 0 or 10 dpi (**Fig 2**).

### Histopathology and immunohistochemistry (IHC)

**Fig 3:**
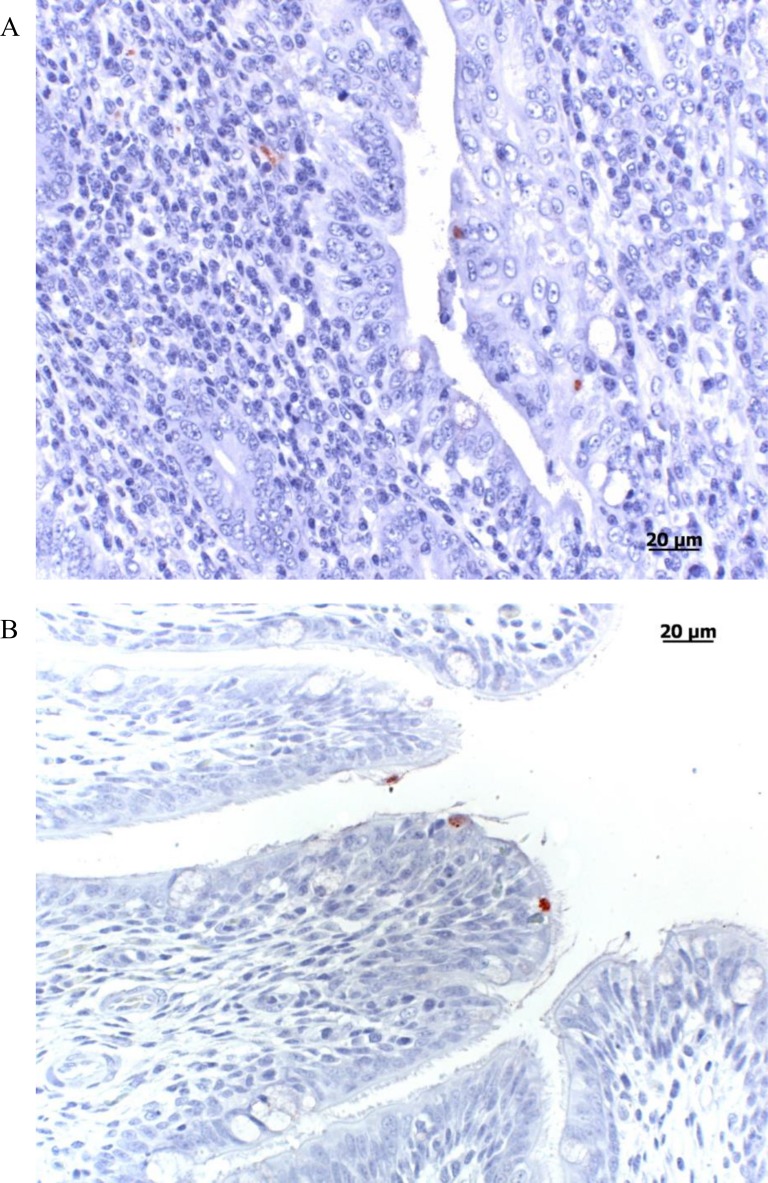
Chlamydial inclusions in affected organ tissue visualized by immunohistochemistry. A. Cecum, contact duckling group 1 (4.3 x 10^8^ IFU/mL). Immunohistochemistry, positive labeling of chlamydial inclusions in the cecal epithelium and sub-epithelial tissue. B. Lung, contact duckling group 1 (4.3 x 10^8^ IFU/mL). Immunohistochemistry revealed chlamydial inclusions in the ciliated epithelium of a bronchus (AEC/peroxidase method, hematoxylin counterstain).

Sections from the spleen, lung and cecum of the seven inoculated and seven contact birds in Group 1, and the seven control birds (Group 4) taken on 10 dpi were examined by histopathology and IHC. Hematoxylin and eosin staining revealed no lesions in the tissues (spleen, lung, and cecum) from all these animals. By IHC, a few chlamydial inclusions were found in tissues from 2/7 infected and 6/7 contact birds (**Table 1**). Five contact birds had a few positive cells in the spleen and/or lungs whereas 4/6 had some positive cells in the cecum. The lung sample for bird no. 6 from the infected group was positive, with one inclusion detected in the bronchus epithelium (**Fig 3**) and the cecum, whereas bird no. 2 had only a few positive cells in the cecum. Four consecutive serial sections of the spleen, lung and cecum of two contact birds (Numbers 3 and 4) from Group 1 were examined in more detail (data not shown). In both animals, one to 15 positive cells per section could be observed in the cecal epithelium and sub-epithelial tissue in at least one serial section. Bird no. 3 also had a single positive cell in the spleen and lung in at least one section, whereas the other bird was negative in all spleen and lung sections (**Fig 3**).

**Table 1:** IHC results from sections of the spleen, lung and cecum of the 7 inoculated and 7 contact birds of Group 1, and of the 7 control birds (Group 4) taken on 10 dpi.

	Group 1 (inoculated)							Group 1 (contact)							Group 4 (control)						
Bird Id	1	2	3	4	5	6	7	1	2	3	4	5	6	7	1	2	3	4	5	6	7
spleen	-	-	-	-	-	-	-	+	+	-	-	-	+	+	-	-	-	-	-	-	-
lungs	-	-	-	-	-	+	-	+	-	+	-	-	-	-	-	-	-	-	-	-	-
cecum	-	+	-	-	-	+	-	+	nd	-	-	+	+	+	-	-	-	-	-	-	-

nd: not done, +: detection of at least one inclusion in one section, -: no inclusion detected

### Shedding of chlamydiae

The examination of cloacal swabs from all 4-day-old ducklings before inoculation showed a low-level presence of *C*. *psittaci* in 11 out of 14 birds (mean Cq = 36.3). Partial sequencing of the *omp*A gene from one of these positive cloacal swab samples revealed a sequence identical to the inoculated strain (genotype E/B sub-genotype 06–859, accession number: EU159263.2).

Cloacal swabs from control birds on 10, 28 and 84 dpi were all negative (data not shown).

For the challenged groups, the level of cloacal shedding was correlated to the bacterial load detected in their organs. The shedding curves given in **Fig 2 I** revealed maximum values of chlamydial genome copies on 7 dpi for Groups 1 and 2, and on 10 dpi for Group 3.

### Strain re-isolation and molecular characterization

To assess the viability of the *C*.*psittaci* detected by PCR, all ceca collected on 10 dpi from Group 1 were inoculated onto embryonated chicken eggs. Isolates were successfully recovered from seven ceca (four inoculated and three contact birds) after one passage (**Table 2**). The *ompA* gene sequences of these isolates were identical to those of the challenge strain (data not shown).

**Table 2:** Strain re-isolation and molecular characterization results. All ceca collected on 10 dpi from Group 1 were inoculated onto embryonated SPF chicken eggs. The *ompA* gene sequence of each isolate was determined.

	Number of birds	Re-isolation	*omp*A related genotype
Group 1 (inoculated)	7	4/7	E/B sub-genotype 06–859
Group 1 (contact)	7	3/7	E/B sub-genotype 06–859

## Discussion

Previous studies on ducks indicated that *C*. *psittaci* infections were mostly asymptomatic in the field [14,15], and the fecal-oral route has recently been assumed to partly explain bird-to-bird transmission and the endemic nature of *C*. *psittaci* on duck farms [14,15]. The present oral infection model was designed to reproduce the probable main route of infection in the field through the ingestion of *C*. *psittaci*-infected materials, including contaminated drinking water, food and/or pastures. Apart from minor transient symptoms in Group 1, neither clinical signs nor macroscopic or histopathological lesions were observed in orally-infected animals throughout the present study, thus suggesting development of an asymptomatic infection. At the same time, examination of the internal organs revealed systemic dissemination of the challenge strain in the birds, irrespective of the inoculation dose. Heterogeneous bacterial dissemination in different organs was observed in each infected group. The highest bacterial loads were detected in the spleen, liver and the small and large intestinal tracts, with the cecum being the most highly-infected organ each time, regardless of the inoculation dose or time point. Similar observations were made for chickens orally infected with *Salmonella enteritidis*, with the spleen, liver and cecum colonization reaching its maximum level during the first week post-inoculation [21,22] and the cecum being the most highly-colonized organ. In an experimental model involving piglets orally inoculated either with *C*. *suis* or *C*. *psittaci* isolates, productive enteric infections were generated and systemic bacterial dissemination was also observed, the highest bacterial loads being similarly detected in the small intestine [18]. Chlamydiae reside in the gastrointestinal tract of virtually all natural hosts, where they can persist for a long time without any overt inflammation or pathology [21, 22, 23]. The short duration of the present study does not allow to predict how the infection would progress, nor to determine if organ colonization by *C*. *psittaci* is transient or persistent. Data from field studies showed a general downward trend of shedding levels over time, which remained low till the end of the breeding process [4,15]. In *Salmonella*-infected chickens, spleen and liver colonization lasted for about three weeks while cecum colonization persisted over ten weeks [21,22]. In mice orally inoculated with *C*. *muridarum*, the lower intestinal tract was colonized and the mice were unable to clear the infection [23]. There are no comparable data for *C*. *psittaci*-infected ducks, which underlines the need for further studies on dissemination kinetics and pathogen persistence.

The IHC analysis of samples from birds infected with the highest dose (Group 1) showed very few chlamydial inclusions in the examined samples. The differences between the findings of PCR and IHC can be explained by the IHC’s lower sensitivity, as only inclusions can be detected by the latter rather than individual EBs and RBs or DNA copies, and also by the fact that tissue samples examined by PCR and IHC are not exactly the same.

Prior to inoculation, 11/14 sacrificed birds were identified as low *C*. *psittaci* shedders. Ducklings included in this study came from a naturally raised flock (non-SPF) and might have been previously infected by vertical transmission [24], although no shedding in female ducks had been detected at laying time. At 14 days of age, ducklings from the control group were all PCR negative (organs and cloacal shedding), thus suggesting an early and self-limited infection with spontaneous clearance. These findings were confirmed by follow-up checks carried out on cloacal swabs from control birds (n = 11) aged 4 and 12 weeks and kept in BSL1 after the end of the experiment (data not shown). The initial chlamydial load detected in the 4-day-old ducklings before the start of the experiment was therefore not sufficient to result in development of an active infection and/or carriage.

Interestingly, by the end of the experiment, the infection level of contact birds was similar to that of the inoculated birds in their respective groups. Horizontal transmission to other birds appears to have occurred in a short time period (less than 10 days) e.g. through direct contact with infected carriers, ingestion of fecally-contaminated material or airborne transmission, as described for *Salmonella* transmission in chicks [25–27].

Avian chlamydiosis is frequently associated with respiratory symptoms and mortality [5,28], as reported in infected turkeys [10,29] and psittacines [30], whereas infections in ducks appear to be mostly asymptomatic [14,15]. *C*. *psittaci* is quite a heterogeneous species, with some avian genotypes appearing to occur more often in a specific order of birds [28] and with *in vitro* and *in vivo* differences in virulence reported among genotypes [31,32]. Experimental infections involving various *C*. *psittaci* genotypes were shown to produce respiratory symptoms in birds after aerosol challenge [9,33,34]. In our experiment, the oral administration of an E/B *C*. *psittaci* strain to ducklings led to an asymptomatic infection with the bacteria detected in all targeted organs. The selected *C*. *psittaci* strain used in our study was isolated from an infected but asymptomatic duck, while severe human cases of psittacosis occurred on this farm [15]. The inoculation route plays an essential role in the clinical outcome.

Furthermore, in birds naturally infected with *C*. *psittaci* and displaying clinical signs, a number of field investigations revealed the presence of co-infecting agents, such as *Ornithobacterium rhinotracheale* in chickens [35], circovirus in pigeons [36] or, more recently, a newly discovered adenovirus in *C*. *psittaci*-infected psittacines [37]. Exacerbation of the clinical course due to co-infecting *C*. *avium* or *C*. *gallinacea*, two new avian species recently described [3], is also conceivable in birds, but has yet to be demonstrated. As the impact of such co-infections on *C*. *psittaci* carriers still needs to be studied in depth, the present model can also be useful in this context.

In conclusion, the oral inoculation of ducklings with a *C*. *psittaci* isolate resulted in asymptomatic infection, yet *C*. *psittaci* dissemination in tissues was systemic and linked to high fecal shedding. This animal model may be used to evaluate therapeutic schemes and vaccines to reduce the excretion level of ducks in order to limit the exposure of humans, farm animals and possibly even wildlife.
